# Megaherbivore coprolite DNA: yields and comparison of three ancient DNA extraction protocols on coprolites of giant ground sloth *Mylodon darwinii*

**DOI:** 10.7717/peerj.21009

**Published:** 2026-06-12

**Authors:** Maria H. Zicos, Ian Barnes, Laurent Frantz, Selina Brace

**Affiliations:** 1 Department of Science, Natural History Museum, London, United Kingdom; 2 School of Biological and Behavioural Sciences, Queen Mary University of London, London, United Kingdom; 3 Faculty of Veterinary Medicine, Ludwig-Maximilians-Universität München, Munich, Germany

**Keywords:** Ancient DNA, Coprolites, PCR inhibition, *Mylodon darwinii*

## Abstract

Coprolites offer rich potential for palaeodietary studies as snapshots of past dietary behaviour and environment. They require adapted laboratory methods to retrieve the DNA of the depositor, its microbiome, diet and environmental taxa. Here we compare the performance of three common ancient DNA (aDNA) extraction methods to recover metagenomes from coprolites of Darwin’s ground sloth *Mylodon darwinii* from Cueva del Milodón (Chile). The Qiagen PowerSoil Kit outperformed the other two methods in terms of DNA recovery and library complexity, but the communities inferred from the DNA extracted by the three methods were similar. We were able to recover signatures of local Patagonian flora, as well as sloth mitochondrial genomes, confirming the taxonomic identity of the coprolite depositors.

## Introduction

Among the preserved tissues and materials from which degraded biomolecules such as DNA can be recovered, are coprolites. These are faecal remains that become preserved either as fully distinguishable units or aggregated and/or compressed and combined into soil. They are found throughout the fossil and subfossil record, from spiralised coprolites in the Cambrian (c. 500 million years ago), to more recent finds such as the Viking-age Lloyds Bank Coprolite in York (UK; Jones, 1983; Hunt et al., 2012). Younger faecal material that is not yet fossilised is sometimes called palaeofaeces to distinguish from mineralised specimens such as dinosaur coprolites (*e.g.*, Green & Speller, 2017; Hagan et al., 2020).

Preserved coprolites provide a snapshot of the depositing animal’s diet, health, parasites, microbiome and environment (*e.g.*, Boast et al., 2018; Oyarzún-Ruiz et al., 2021). Their composition can be analysed morphologically and/or using molecular techniques, though when the preserved parasitic or environmental taxa are non-diagnostic, such as remains in the plant family Poaceae (*e.g.*, Moore, 1978; Markgraf, 1983; Van Geel et al., 2022), molecular methods may be preferable, subject to the availability of reference material. Coprolite time series can provide insights into the changing health and diet of a population (Søe et al., 2018; Tams et al., 2018). In historical/ancient microbiome studies, where gut tissue preservation is rare, coprolites are often the best or only proxy available to explore the gut microbiome. In diet investigations, morphological analysis of toothwear (*e.g.*, Tütken et al., 2013; Kalthoff & Green, 2017) and stable isotope analysis (*e.g.*, Bonin et al., 2020; Tejada et al., 2021) are useful indicators, but coprolites, which represent the hours to days following food consumption, provide a finer temporal and taxonomic level resolution (*e.g.*, Witt et al., 2021).

Coprolite analysis also provides information about the palaeoenvironment of the depositor, for example through wind-borne pollens (*e.g.*, Markgraf, 1985; Moore, 1978). While the seasonal timing of coprolite deposition is generally unknown, plant remains may provide clues as to the season of deposition (*e.g.*, inflorescences and seeds in Van Geel et al., 2022).

Although rich in DNA information, coprolites contain chemical inhibitors of PCR reactions, for example in the form of humic acids and Maillard reaction products (*e.g.*, Poinar et al., 1998). Inhibitors interact with enzymes involved in library preparation prior to sequencing, such as PCR amplification, reducing DNA yields even in the current era of High Throughput Sequencing. These chemical inhibitions resulted in the development of dedicated commercial and in-house protocols for extraction from soil, faeces and coprolites (*e.g.*, Qiagen PowerSoil kit, Poinar et al., 1998; Hagan et al., 2020). Removal of other tissue-specific inhibitors is also necessary for other material, such as lignin in plants, for which dedicated protocols were also designed (*e.g.*, the cetyltrimethylammonium bromide method per Doyle & Doyle (1987) and Qiagen Plant Mini Kit). While coprolite research is now well-established in the ancient DNA (aDNA) community (*e.g.*, Boast et al., 2018; Søe et al., 2018; Tams et al., 2018), there have been limited studies formally comparing the recovery of DNA with different extraction methods (Hagan et al., 2020). Here, we compare the aDNA recovery (in particular plant DNA) of three popular extraction methods in coprolites of an extinct Late-Pleistocene megaherbivore, to study consumption and environmental signals.

Darwin’s ground sloth *Mylodon darwinii* (Owen 1839) was one of at least eight species of large terrestrial sloths found in southern South America in the Late Pleistocene (Pujos, De Iuliis & Cartelle, 2016). Approximately one to two tons in weight (Christiansen & Fariña, 2003), it became extinct approximately 10,000 calibrated years before present (BP) (cal yr BP; Villavicencio et al., 2016) and is best known for well-preserved skins, bones and coprolites found in Cueva del Milodón (Ultima Esperanza, Chile) from 1895 (Nordenskjöld, 1900; Hauthal, Roth & Lehmann-Nitsche, 1899). This site’s record begins around 18,000 years BP, and includes Pleistocene fauna and later human occupation (*e.g.*, Nordenskjöld, 1900; Saxon, 1976). While human presence was recorded at the cave from approximately 13,000 cal yr BP (see Villavicencio et al., 2016), there is currently no evidence for sloth-human interactions in the area (Borrero & Martin, 2012). Examination of plant remains in *M. darwinii* coprolites at Cueva del Milodón revealed a grazing diet (Moore, 1978; Markgraf, 1985), contrasting evidence of a selective diet with browsing in other parts of its range (Bargo, Toledo, and Vizcaíno, 2006; Varela et al., 2023). Paleoenvironmental records of the area indicate a vegetation shift from cold steppe to southern beech (*Nothofagus*) forests around 12,000 cal yr BP (Markgraf, 1985; McCulloch et al., 2021), which would have had a large impact on grazing communities. The extensive record of *M. darwinii* coprolites in Cueva del Milodón provides the opportunity to assess shifts in their diets as their environment changed with a warming climate.

Here we start exploring signals of this species’ diet and environment through aDNA analysis, also addressing issues of reference biases and previous concerns on the current lack of power of metagenomic analysis to recover signals of *Mylodon* DNA or Patagonian flora in similar material (Van Geel et al., 2022). This study demonstrates the rich potential of *Mylodon darwinii* coprolites for metagenomic analysis in the future with suitable research design and analysis software.

## Materials & Methods

For all sections, more detailed information can be found in the Supplemental Information 1.

### Samples

Seven specimens from the NHMUK fossil mammal collection were sampled for this study. Two specimens were full coprolites collected at Cueva del Milodón in the early 20th century with no other context information. The other five were excavated from a layer of compacted *Mylodon* dung at Cueva del Milodón in 1976 (Saxon, 1976) which can reach up to one m in depth (Hauthal, Roth & Lehmann-Nitsche, 1899; Nordenskjöld, 1900). The five samples were selected to represent the top, bottom, and three evenly spaced points in between for this layer (layer 3 in Saxon’s trench 5; Fig. S1).

### Sampling and radiocarbon dating

Sampling and subsequent aDNA work was conducted in the NHMUK ancient DNA laboratory. Samples from the 1976 excavation were sampled following Wood & Wilmshurst (2016), and a modification of this was used on the two full coprolites to limit destruction. Specimens were subsampled three times according to extraction method weight requirements.

Sampling for radiocarbon dating was performed similarly with a sample weight aim of 1 g and sent to ORAU for Accelerator Mass Spectrometry (AMS) radiocarbon dating (Brock et al., 2010). Date calibration was conducted in Oxcal online (v. 4.4.5; Bronk Ramsay, 2021; Hogg et al., 2020).

### Extractions

For each sampled specimen, three extractions were carried out to address concerns of inhibition in coprolites: (1) an established aDNA protocol specialised for the recovery of small fragments (Dabney et al., 2013)(modified as in Brace et al. (2019)), (2) the Qiagen DNeasy Plant mini kit, and (3) the Qiagen DNeasy PowerSoil kit, designed to maximise DNA recovery from plant material and microorganisms in soil/faeces, respectively (both purchased 2018). These kits were selected based on use in aDNA studies for removal of inhibitors in soil/coprolites (*e.g.*, Boast et al., 2018) and plant remains (*e.g.*, Li et al., 2016; Wales et al., 2019), to study diet and environmental signals of this herbivore. These protocols were slightly modified as follows: (a) the Dabney et al. (2013) protocol with viral HighPure silica columns (after Brace et al., 2019) was further adapted for lysis of tissue with Qiagen ATL buffer and proteinase K, and a 24 h incubation at 56 °C with rotation (now referred to as Dabney Tissue). (b) The DNeasy Plant mini kit extractions were incubated to two hours with rotation following Wales et al. (2019). (c) In the PowerSoil protocol, mechanical disruption was conducted through one minute of vortexing, then incubated at room temperature for 20 min with rotation. Extracts were quantified on a qubit 2.0.

### Libraries and sequencing

Double-stranded libraries were prepared following Meyer & Kircher (2010), then quantified on an Agilent TapeStation 2200 using a D1000 tape and reagents. Shotgun libraries were sequenced on a NextSeq 500 at the NHMUK sequencing facility, with a mid-output 2 × 75 bp run. Library aliquots were sent to DAB for hybridisation capture of the mitochondrial genome, performed in 6 double reactions following Delsuc et al. (2019), then sequenced on a Novaseq S4 2 × 150 bp run.

### Data preprocessing

Raw reads were quality-checked with FastQC (http://www.bioinformatics.babraham.ac.uk/projects/fastqc), trimmed and merged using adapterRemoval v. 2.2.2 (Schubert, Lindgreen & Orlando, 2016), and duplicate reads were removed using Prinseq (Schmieder & Edwards, 2011).

### Alignments to sloth

Trimmed and merged reads from shotgun and captured libraries were mapped to references using BWA (v. 0.7.17-r1188; Li & Durbin, 2009). The closest living relative of *Mylodon*, the southern two-toed sloth *Choloepus didactylus* (accession GCA_015220235.1), was used as a reference nuclear genome. The mitochondrial genome of *Mylodon darwinii* (Delsuc et al., 2018; accession NC_037941.1) was used as a reference for mitochondrial analysis. Mapping was performed with bwa aln, and quality filtering with SAMTOOLS (v. 1.12; Li et al., 2009). Coverage depth and damage estimations were conducted with Qualimap (v. 2.2.2a; Okonechnikov, Conesa and García-Alcalde, 2016) and MapDamage (v. 2; Jónsson et al., 2013), respectively.

### Taxonomic classification

Trimmed and merged shotgun reads were mapped to the NCBI nt database with ncbi-blast+ (v. 2.9.0-2; Camacho et al., 2009), these outputs were run through PIA (Cribdon et al., 2020), then analysed in R (v. 4.2.3; R Core Team, 2023) using packages taxonomizr (v. 0.10.2; Sherrill-Mix, 2023), phyloseq (v. 1.40.0; McMurdie & Holmes, 2013) genefilter (v. 1.78.0; Gentleman et al., 2022) and ggplot2 (v. 4.0.1; Wickham, 2016).

Community composition was summarised through non-metric multidimensional scaling (NMDS) in vegan (v. 2.7-2; Bray & Curtis, 1957; Kruskal, 1964; Oksanen et al., 2022). Alpha diversity metrics of richness and evenness (Shannon & Weaver, 1949; Simpson, 1949), were compared through Kruskal–Wallis tests (Kruskal & Wallis, 1952). Significant differences were further analysed with pairwise Wilcoxon rank-sum tests with multiple comparison correction (Wilcoxon, 1945; Dunn, 1961; Benjamini & Hochberg, 1995) and effect size estimation (Tomczak & Tomczak, 2014).

### Taxonomic classification verification

A list of vascular plant species was compiled to represent known species near Cueva del Milodón (Moore, 1978) and between 52° and 56°S in Chilean Patagonia (Pisano, 1977) to account for changes in plant communities through time. The taxonomy was verified and updated to match that of the Basic Local Alignment Search Tool-Protein Interaction Analysis (BLAST-PIA) pipeline. BLAST-PIA Taxonomic assignments were compared against this plant list to determine whether they were likely to represent local flora, both using the main taxonomy Table in R and leveraging further information from the taxid number.

### Properties of the generated data

To study the characteristics of the different extractions on our extracts and libraries, the following metrics were compared: (1) Extract concentration (representing DNA recovered at extraction), (2) library concentration after amplification (representing realised DNA yield), (3) sequenced read lengths, (4) library complexity (representing proportions of unique molecules in the sequenced libraries), (5) proportion of identified reads, and finally (6) proportion of identified plant reads.

Additionally, to check for short DNA fragment recovery between the different silica columns, average fragment length, molarity and proportion of the sample were compared for the 155–180 bp region of the TapeStation traces, comparing them to the main sample 180–400 bp region (Fig. S3).

Finally, the effects of sample type, extraction and sample age on the metagenomic community were explored through Mantel tests and Analysis of Similarities (ANOSIM) tests, respectively (Legendre & Legendre, 2012; Clarke, 1993; Warton, Wright & Wang, 2012).

## Results

### Radiocarbon dating

All samples yielded radiocarbon dates, ranging from 16,276 cal yr BP (95% CI [16,868–15,745] cal yr BP) to 14,627 cal yr BP (95% CI [14,947–14,316] cal yr BP; Table S1, Fig. S2). The AMS measurements indicate that this layer of compressed dung, representing the core occupation of the cave by *Mylodon darwinii*, was deposited over approximately 1,600 years. The two full coprolites (NHMUK PV M102296 and 102,299) also belong to this time interval.

### DNA concentration

**Figure 1:**
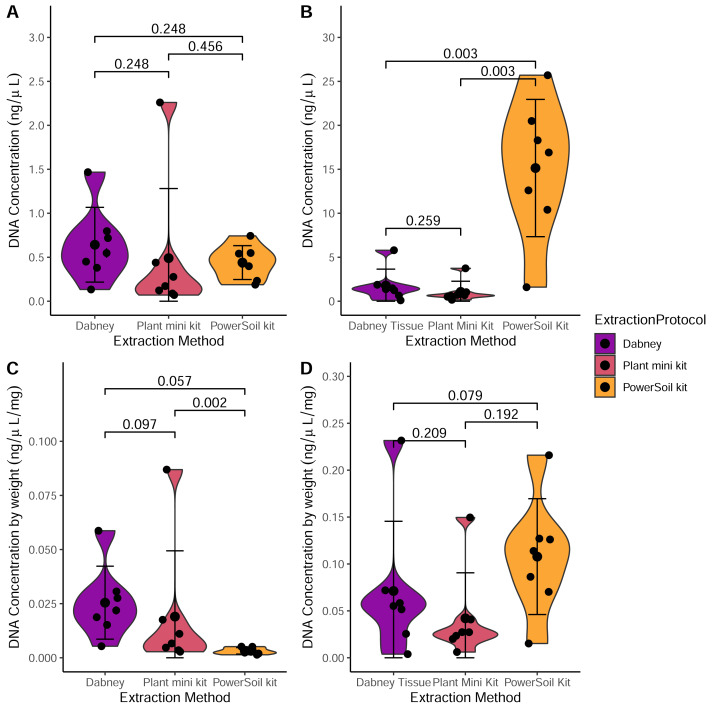
Extract and library concentrations per extraction protocol in coprolites of *Mylodon darwinii*. (A) DNA concentration in extracts. (B) DNA concentration in libraries. (C) Extract DNA concentration per mg starting sample. (D) Library DNA concentration per mg starting sample. Wilcoxon Rank Sum test *p*-values are displayed, with Benjamini–Hochberg adjustments for multiple comparisons.

DNA concentration in extracts ranged 0.07–2.26 ng/µL or 0.0014–0.087 ng/µL/mg sample (overall concentration mean µ= 0.524 ng/µL, standard deviation *σ* = 0.497 ng/µL; scaled concentration µ= 0.01589 ng/µL/mg, *σ* = 0.0208 ng/µL/mg; Fig. 1; Table S3). Extracts with visible colouration registered the highest DNA concentrations. Raw extract DNA concentrations did not significantly differ between extraction protocols (*p* = 0.14), but when scaled by sample weight, a significant difference was detected (Kruskal–Wallis chi-squared *χ*^2^ = 11.629, *df* = 2, *p* = 0.00298, effect size = 0.535), with the PowerSoil kit extracts yielding the lowest concentrations per sample weight and being found statistically lower than the Dabney extracts (Wilcoxon Test statistic *W* = 41, adjusted *p* = 0.002).

DNA yields in finished libraries varied significantly between extraction methods (Kruskal–Wallis *χ*^2^= 7.9555, *df* = 2, *p* = 0.01873; effect size = 0.313), with PowerSoil kit library concentrations greater than either the Dabney tissue protocol or Plant mini kit (no significant difference was found between the latter two; Wilcoxon *W* = 35, adjusted *p* = 0.627; Fig. 1; Table S3). When scaled by sample weight, the PowerSoil kit concentrations were still higher but no longer significantly differed from other protocols (Kruskal–Wallis *χ*^2^ = 5.2171, *df* = 2, *p* = 0.07364).

Specimen NHMUK PV M103818 (bottom of the trench layer) tended to fail recovery of DNA, yielding concentrations comparable to controls for both the Dabney Tissue and Plant kit (samples MZ042 and MZ049). Additionally, the extracts were coloured darker and resulted in high Qubit readings. However, this specimen was successfully amplified with the PowerSoil kit. In contrast, trench specimen NHMUK PV M103823 yielded little DNA when extracted with the PowerSoil kit (sample MZ024), performing well with other kits (samples MZ043 and MZ050).

### Fragment size and read length

In libraries, the short insert size region of 155–180 bp in the TapeStation traces, selected to represent short reads of length <35 bp, corresponded to 0.27–31.5% of the sample traces. No difference was identified between extraction methods in the average read length or proportion of the library contained in that region (Kruskal–Wallis *χ*^2^ = 4.62, *df* = 2, *p* = 0.0992 for average read length; Kruskal–Wallis *χ*^2^ = 4.39, *df* = 2, *p* = 0.111 for percent of library in region). The region molarity differed between extraction types (Kruskal–Wallis *χ*^2^ = 10.5, *df* = 2, *p* = 0.00514, effect size *H* = 0.474), in particular with the PowerSoil kit showing larger molarity than the other two (Wilcoxon pairwise comparison test with continuity correction W: Dabney *vs* Powersoil kit *W* = 6.5 and adjusted *p* = 0.038, Plant *vs* PowerSoil kit *W* = 2 and adjusted *p* = 0.007; Fig. S3) indicating a higher quantity of short reads.

Up to 14.4 million raw paired reads per library were generated (Table S3). Trimmed and merged reads ranged from 25 bp to 141 bp in length (see supplementary methods for filtering parameters). Average read lengths ranged 49.5–85.9 bp per sample, with overall mean read length of 70.4 bp (*n* = 58.34 million reads excluding controls).

**Figure 2:**
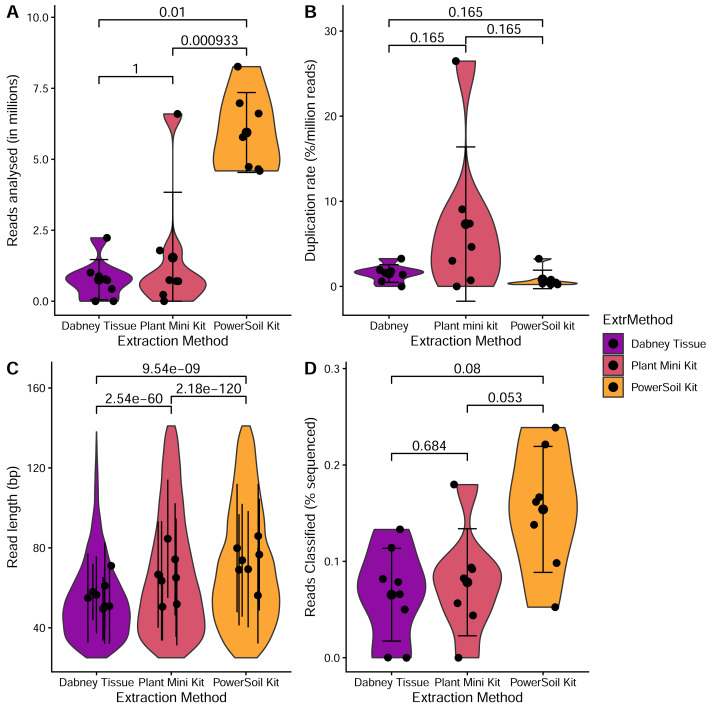
Sequenced read statistics of the *Mylodon darwnii* libraries generated from coprolites and Cueva del Milodón Trench 5 samples in this study, by extraction method. (A) Reads used in analysis, after trimming and merging paired end raw reads. (B) Duplication rate in analysed reads. (C) Average read length of analysed reads. (D) Reads classified as percentage of analysed reads. Wilcoxon Rank Sum test *p*-values are displayed for the pairwise comparisons, with Benjamini–Hochberg adjustments for multiple comparisons.

Average read length differed between extraction methods, with µ= 53.4 bp in Dabney Tissue, µ= 66.3 bp in the Plant kit, and µ= 74 bp in the PowerSoil kit extracted libraries (Kruskal–Wallis rank sum test *χ*^2^ = 3038158, *df* = 2, *p* < 2.2e−16; Sample sizes: Dabney reads *n* = 6,065,859 reads, Plant kit reads *n* = 10,764,775; Soil kit reads *n* = 41,599,590 reads; Fig. 2C).

A second set of tests was conducted on subsets of reads to account for the different sample size of reads across extraction methods, or between coprolite and trench samples (see Supplementary Methods). The above results were replicated: read lengths were different between extraction methods (subset *n* = 9,000 reads, 500 per library, Kruskal–Wallis *χ*^2^ = 629.28, *df* = 2, *p*-value < 2.2e−16) and between sample types (*n* = 2,000, 1,000 reads per sample type; Wilcoxon: *W* = 418,898, *p* = 3.365e−10). When testing read length between specimens (*n* = 3,500, 500 reads per specimen), not all samples had significantly different means from each other.

### Library complexity

The raw duplication rate of libraries (in percent duplicate reads) ranged from 1.34% to 6.7%. Library MZ024 from the PowerSoil kit (NHMUK PV M103823) yielded a very small concentration compared to the rest of the Powersoil replicate libraries and its duplication rate was comparable to the controls (Table S3). This sample was excluded from the protocol comparisons.

For raw duplication rate, marginal support was found for different duplication levels between the Qiagen PowerSoil and Qiagen Plant mini kits (*W* = 35, *p* = 0.0513). This evidence for differences increases when accounting for sequencing effort (*W* = 39, *p* < 0.01). When including the low-success rate Dabney Tissue libraries into the relative duplication rate, there was indication of a difference between all extraction methods (sometimes marginal), with lowest average rate for the Powersoil kit, followed by the Dabney extractions and finally the Plant mini kit (Kruskal–Wallis rank sum test *χ*^2^ = 11.825, *p* = 0.002706; pairwise Powersoil *vs* Plant kit: *W* = 39, *p* = 0.008159; pairwise Powersoil *vs* Dabney Tissue: *W* = 35, *p* = 0.004329; pairwise Plant *vs* Dabney: *W* = 6, *p* = 0.06494; Fig. 2B).

### Host DNA - authentication

The shotgun libraries yielded 0-19 reads mapping to the mitochondrial genome of *Mylodon darwinii* (Table 1; Table S3) but six captured libraries had > 10 reads aligning for coverages ≥ 0.2x, with four reaching > 1x (max = 6.1x; Table 1). Library alignments to the nuclear genome of Linnaeus’ two-toed sloth *Choloepus didactylus*, yielded 0.04–1.27% endogenous proportions (Table S3).

**Table 1:** Final reads mapping to the mitochondrial genome of *Mylodon darwinii*. The results are shown first for shotgun libraries by extraction method, displaying number of unique reads mapping to the reference (accession NC_037941.1) with mapping quality threshold of 30. The best mapping libraries for each specimen were selected for mitochondrial capture, excepting Specimen NHMUK PV M 103823 for which both the PowerSoil and Dabney libraries were captured). Captured library results are as followed: final reads mapping at MQ30, enrichment fold as compared to the best shotgun library for the sample, and final coverage depth of the mitochondrial genome.

Collection numbers (NHMUK)	Sample type	Shotgun libraries			Captured libraries		
		Dabney tissue	PowerSoil kit	Plant mini kit	Final reads	Enrichment ratio	Coverage (x)
PV M103822	Saxon 1976	0	5	1	1,371	274	6.1
PV M103821	Saxon 1976	0	0	1	103	103	0.3
PV M103824	Saxon 1976	0	0	0	7	inf	0.0
PV M103818	Saxon 1976	0	11	0	702	58.5	2.2
PV M103823	Saxon 1976	0	0	0	2; 68	inf	0.2
PV M102296	whole coprolite	1	19	0	517	27.2	1.6
PV M102299	whole coprolite	11	7	0	675	96.4	2.2
Extraction Control	Sterile water	0	0	0	NA	NA	NA
Library control	Sterile water	0	0	0	NA	NA	NA
Average (samples)		1.7	6.0	0.3	482.4	114.1	1.8
Standard Deviations (samples)		4.1	7.1	0.5	496.1	110.1	2.1

### BLAST–PIA identifications and ecological analysis

240 taxa were identified from 81,634 reads in the whole dataset (0.14% of 58,451,316 reads analysed). Within Streptophyta, 1,353 reads remained for 20 taxa (1.6% of classified reads; 0.002% of the dataset). The number of identified reads was different between extraction methods for all taxa and plant taxa, with more identifications in the PowerSoil kit libraries (Kruskal–Wallis *χ*^2^ = 12.285, *df* = 2, *p* = 0.002149 for all taxa; Kruskal–Wallis *χ*^2^ = 9.9171, *df* = 2, *p* = 0.007023 for plant taxa; Fig. 2D), resulting in higher species richness (Kruskal–Wallis *χ*^2^ = 8.3795, *df* = 2, *p* = 0.01515 for plant taxa; Kruskal–Wallis *χ*^2^ = 10.165, *df* = 2, *p* = 0.006204 for taxa).

However there was no difference in species evenness between the extractions, either with the Shannon-Weaver index (Kruskal–Wallis *χ*^2^ = 1.7941, *df* = 2, *p* = 0.4078 for all taxa; Kruskal–Wallis *χ*^2^ = 3.6986, *df* = 2, *p* = 0.1573 for plant taxa) or the Simpson Index (Kruskal–Wallis *χ*^2^ = 0.77886, *df* = 2, *p* = 0.6774 for all taxa; Kruskal–Wallis *χ*^2^ = 0.45656, *df* = 2, *p* = 0.7959 for plant taxa). This indicates that the evenness of abundances of identified taxa did not change with increases in species richness (Fig. S4).

**Figure 3:**
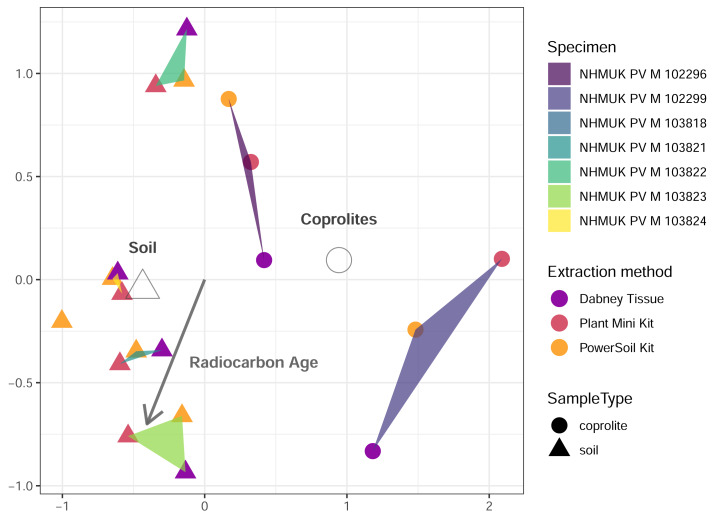
NMDS (Bray–Curtis distance) of the metagenomes in the *Mylodon darwinii* coprolite libraries. The centroids for the effect of sample type on the community structure of the libraries are represented as the empty circle and triangle, the effect of Radiocarbon age on community composition is shown by the arrow (envfit scores, vegan package), and the grouping of the extraction replicates of each samples by the polygons in the purple-green-yellow colour scale. Extraction method was not found to have a significant effect on community structure when accounting for sequencing depth (ANOSIM test statistic = −0.0866, *p*-value=0.874).

The community compositions broadly overlapped with regards to extraction protocol (Fig. 3). There was strong evidence for an effect of radiocarbon age on community composition (Mantell test, r statistic = 0.7395, *p*-value = 1 × 10^−4^), indicating the samples themselves drew differences. Extraction method did not seem to influence the community composition of the samples (ANOSIM test statistic = −0.0866, *p* = 0.874), however there was evidence for a difference in community composition between the full coprolites and the samples from the Saxon trench coprolite layer in the cave stratigraphy (ANOSIM test statistic = 0.2837; *p* = 0.168).

### Local plant list and taxonomic verification

The plant list based on Pisano (1977) and Moore (1978) comprised 1,741 records of plants in 34 assemblages categorised in four plant communities: Patagonian steppe, Deciduous Magellanic Forest, Evergreen Magellanic Forest, and Magellanic Tundra. 206 genera and 381 unique species were verified for current nomenclature (Table S4).

**Figure 4:**
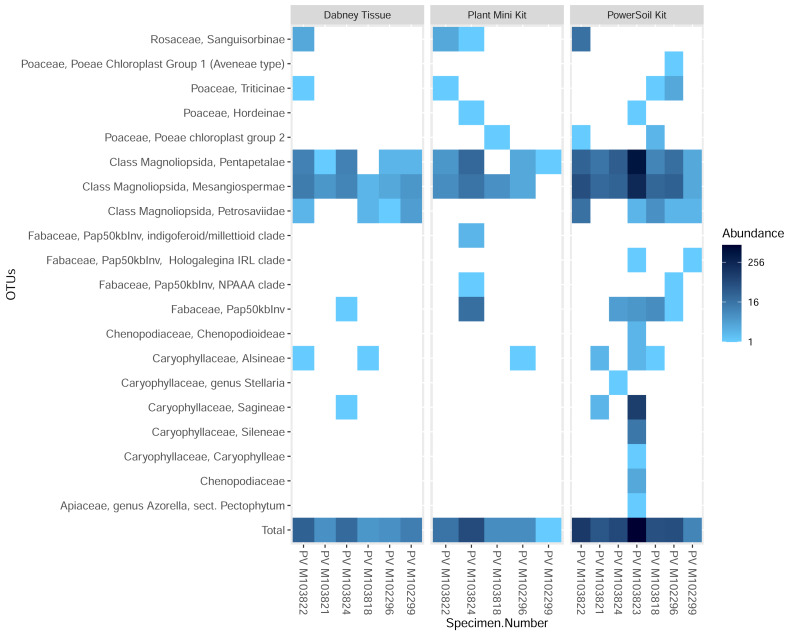
OTU information and abundance of plant reads detected in each sample with the BLAST-PIA pipeline. Note: Pap50kbInv = Papilionoideae 50 kb. The Total row represents totals per library. More detailed information is available in Table S4.

Of the 20 Streptophyta OTUs were identified (Fig. 4), 17 went to Family level, but the last three were only classified to class Magnoliopsida, providing little information on identity. Families identified were, in order of read abundances: Caryophyllaceae, Fabaceae, Rosaceae, Poaceae, Chenopodiaceae, and Apiaceae. They are all present in the Patagonian plant list and additional taxid information is shown in Fig. 4 and Table S4. Genus-level identifications occurred for two Operational Taxonomic Units (OTUs), *Azorella* (Apiaceae) and *Stellaria* (=*Moneuria*, Caryophyllaceae), both part of the Patagonian steppe community. Of the other 15 OTUs, all but two groups (“Poaceae, Tricitinae” and “Fabacae, Papilionoideae 50 kb inversion clade NPAAA clade-indigoferoid/millettioid clade”) have local species in the plant list, indicating the possibility a local plant signal.

## Discussion

The results above confirm the potential for *M. darwinii* coprolites and soil from Cueva del Milodón for ancient molecular studies as both radiocarbon dates and DNA were recovered. This is in line with previously noted preservation levels at this locality (*e.g.*, Nordenskjöld, 1900), and successful biomolecular work on other sloth material in the cave, such as dating (reviewed in Villavicencio et al., 2016), aDNA (*e.g.*, Taylor, 1996; Clack, MacPhee & Poinar, 2012; Delsuc et al., 2018; Delsuc et al., 2019), and stable isotopes (Tejada et al., 2021).

### Improvements to sample chronology

The seven new AMS measurements update the chronology of the cave’s compressed sloth dung and soil layer. Previous measurements for the bottom and top of the compressed dung layer of the cave were dates BM-1209 and BM-1210B, respectively (Burleigh & Matthews, 1982), which would yield calibrated dates of 14,444 cal yr BP (95.3% CI [16,300–13,505] cal yr BP; BM-1210B, bottom of the layer) to 14,639 cal yr BP (95.4% CI [15,159–14,092] cal yr BP; BM1209 top of the layer). Instead this dung layer, the core part of *M. darwinii*’s presence, is now dated to 16,276 cal yr BP (95% CI [16,868–15,745] cal yr BP) to 14,627 cal yr BP (95% CI [14,947–14,316]). Available AMS radiocarbon dates of coprolites from Cueva del Milodón are within that age range (Fig. S2).

### Assessing DNA recovery

For the purposes of DNA data generation, the PowerSoil kit performed better overall. Although resulting in smaller starting DNA concentrations per starting weight in the extracts (as previously observed by Murchie et al. (2021)), the overall DNA yield was not different from the other extraction methods, and these PowerSoil extracts ultimately yielded higher concentrations from libraries with the same input of extract and number of amplification cycles (twenty) as the other protocols. This improved performance at the library stage suggests better efficiency when removing inhibitors, which interfered more with library yields in the other methods. Although the plant kit also removes inhibitors, in practice the two kits target different molecules: neutralising plant-specific inhibitors, such as lignin, *versus* environmental inhibitors such as humic acids. However, further testing regarding the specificity of inhibition removal across extraction methods would be required to assess this. It should be noted that since this experiment was conducted, Qiagen superseded the PowerSoil kit with the more recent PowerSoil Pro kit. However, the kit’s chemistry is the same, so the inhibitor-removal benefits should be similar.

While this kit requires more starting material, samples in the order of 100–200 mg soil or megafaunal coprolites are comparatively easy to acquire. Given the comparative success of the libraries produced by this kit, we suggest that for such a sample amount, one set of extractions with the PowerSoil kit will yield better libraries than an equivalent number of extractions and libraries with the other protocols, resulting in time and cost efficiency.

PowerSoil-extracted libraries also had longer reads. The Qiagen kits have different silica spin columns than the Viral HighPure column used for the Dabney Tissue extracts. However, as both commercial kits used the same MB spin columns, differences in yields and fragment lengths between them cannot be attributed to membrane chemistry. Additionally, TapeStation trace analysis indicated that both silica column types were able to recover short fragments in the extracts, with no difference in average length for the short read region. Indeed, the molarity comparison in the TapeStation data (Fig. S3) indicates that the PowerSoil kit, while retrieving more DNA overall, also recovered more short-fragment DNA than the other two methods. When accounting for sequencing effort, PowerSoil kit libraries also resulted in the highest proportions of identified reads, possibly due to the longer molecules facilitating taxonomic identification.

Results from the plant mini kit’s libraries indicate that this protocol, while performing well for archaeobotanical studies, is not the best suited for recovering plant DNA in soil or palaeofaeces as starting material, even with the aim of studying plant DNA in coprolites. While the Dabney protocol performs very well for other substrates such as bones and tissue samples in the archaeological and palaeontological record, here it is the least productive method in terms of read length and proportion of classified reads.

As the Qiagen kits are marketed for recovery of DNA from different taxonomic groups, and it was previously found that extraction protocols can bias the taxa recovered from faeces (Wesolowska-Andersen et al., 2014), the community composition in the three sample replicates were tested for shifts caused by extraction protocol. While the PowerSoil kit retrieved more data, community evenness and structure was more affected by the intrinsic properties of the samples than by the extraction protocol, as found by Hagan et al. (2020). Similarly, in multidimensional analysis the sample replicates were more alike to each other (within specimen) than to other replicates extracted with the same protocol. This indicates that there is no considerable bias of extraction protocol choice on ancient metagenome community structure, implying that comparison of data obtained with different extraction methods is not inappropriate.

### Further methodological advancements

This paper aimed to compare three widely used protocols for degraded DNA extraction in the context of coprolites from cave sediments, but in this fast-changing field there are further methodologies to consider. The PowerSoil kit’s performance, for instance, may also be improved upon. Hagan et al. (2020) tested four methods other than the PowerSoil kit on palaeofaeces, and found that all methods were an improvement on its performance. Different modifications on the Dabney method (Hagan et al., 2020) make it difficult to compare their results directly with the current study and the baseline Dabney protocol. Based on these experimental setup differences we argue that our comparison of Dabney and the PowerSoil kit and performances is still valid, but modifications of the Dabney protocol with PowerSoil-like elements such as the mechanical disruptions may be worth pursuing.

This improvement of the PowerSoil kit on the Dabney protocol is also supported by results in another experiment conducted on sedaDNA samples by Murchie et al. (2021). Their coldspin modifications on the Dabney protocol, which precipitates inhibitors overnight, improved concentration yields and taxonomic identifications in barcoding, shotgun and captured libraries. These authors posited the interesting hypothesis that the unmodified PowerSoil kit and Dabney lie at opposites of the spectrum of inhibition removal and ancient DNA retrieval: Dabney, while performing better at ancient DNA removal than PowerSoil, does not adequately remove inhibition, whereas PowerSoil removes inhibition well but can lose ancient DNA. However, because of these issues, both perform comparatively badly in retrieving most of the amplifiable ancient fragments for libraries, whereas Murchie et al. (2021) propose that the coldspin method gets closer to the ideal point of inhibition removal and retention of ancient fragments with the comparatively gentle inhibition removal of the overnight coldspin precipitation. It has also been shown to improve performance in marine sediments (Bourrau et al., 2025) and could therefore also be applied to coprolites. We note however, the variance in inhibition, where the Murchie samples were so highly inhibited that the Dabney protocol (without cold spin) failed to adapt DNA into libraries, compared to our samples where the Dabney protocol, whilst producing a lower yield was still successful for all but one sample. Finally, the technique requires a temperature-controlled centrifuge, a significant investment, not necessarily possible or practical for all laboratories. Our study therefore provides a useful comparison as to whether to apply the basic principles of the Dabney *versus* the Qiagen kit methods without the need for further equipment.

### Palaeogenetic investigations on *M. darwinii* coprolites

Recently aDNA analysis of another *M. darwinii* coprolite from Cueva del Milodón could not recover any endogenous signal in the sloth coprolite (Van Geel et al., 2022). Similarly, very few of our shotgun libraries had any reads mapping to the mitochondrial genome of *M. darwinii*. However, following the recovery of full *Megatherium americanum* mitochondrial genomes from coprolites with hybridisation capture (Delsuc et al., 2019), we applied a similar approach that yielded *M. darwinii* signal in all but one specimen (Table 1). Authentication of the coprolite’s depositing species with endogenous DNA is therefore difficult but possible with investment in hybridisation capture methods. Regarding the lack of sloth reads in specimen NHMUK PV M103824, it is possible that not all the selected pellets in the compressed dung layer were indeed dung, or that this sample was not well preserved.

We used the BLAST-PIA pipeline for this study as PIA was designed to work with incomplete reference databases, using information from the top BLAST identifications to give likely taxonomic classification (Cribdon et al., 2020). This conservative approach yields few identified reads compared with other methods, but the identifications are more robust. The NCBI nt database was used in the absence of dedicated reference databases for Patagonian flora. However, despite the small number of classified plant reads, there were reliable assignments to taxa representing local flora (Pisano, 1977; Moore, 1978).

Previous investigations of plant taxa in Cueva del Milodón coprolites include pollen work by Markgraf (1985), macrofossil investigation by Moore (1978), and a more recent pollen study on a single coprolite by Van Geel et al. (2022). Of these, only Van Geel et al. (2022) provide an AMS radiocarbon date, allowing viable temporal comparison with this study. However, Moore’s (1978) examination of material from the same layer of trench 5 in the (Saxon, 1976) permits a temporal correlation to our study. Given the difference in the new AMS dates of the dung layer of the (Saxon, 1976) trench compared with the mid-1970’s measurements, tightly linking Markgraf’s results from her material dated in the 1950’s–1980’s to the current chronology is impossible without redating, but her observation of a shift from steppe vegetation to forest vegetation in younger material are concordant with both older (Salmi, 1955) and newer works (McCulloch et al., 2021) and remain valid. Recently, a high-resolution pollen dataset with a robust age model from a sediment core at the top of Cerro Benitez, the mount upon which Cueva del Milodón is located, became available as an archive of environmental pollens in the local area from approximately 16,300 to present (McCulloch et al., 2021).

While the 1,353 identifications with PIA that we report here (Fig. 4, Table S4) are too limited a dataset for a full ecological exploration, and we caveat there will be some reference bias to assignments, the conservative PIA results allow us to identify the presence of plant species, which can be compared with previous works. Read numbers in the PIA identifications will be used here for confirmation of presence rather than discussions of abundance. Most identified reads by PIA were in extracts from the PowerSoil kit (1,182 identified reads, *vs* 78 for Dabney and 93 for Plant mini kit; Fig. 4). Caryophyllaceae had the most identified reads, with OTUs in genus *Stellaria*, Alsinae, Saginae, Sileneae and broader Caryophyllae. The Patagonian species for this family are found in both the Patagonian steppe and Magellanic tundra environments (Pisano, 1977). Pollen of *Colobanthus*, *Silene* and undetermined Caryophyllaceae were found in the Van Geel et al. (2022) coprolite. McCulloch et al. (2021) recorded medium pollen abundance of this family in the environmental core throughout their 16,300 to 13,900 cal yr BP timeline. However no Caryophyllaceae macrofossils were reported by Moore (1978) in the layers corresponding to our Saxon trench 5 samples, perhaps indicating that this taxa represents environmental rather than dietary signals through pollen.

Another family identified is Fabaceae, with four OTUs centred on the Papilionoideae 50 kb inversion clade, which contains Patagonian genera *Adesmia*, *Lathyrus* and *Vicia*, found in the Patagonian Steppe and Deciduous Magellanic forest. *Adesmia* pollen, *Vicia* or undetermined Fabaceae were found in low abundances in the Van Geel et al. (2022) coprolite and environmental core (McCulloch et al., 2021). However Moore (1978) did not report any macrofossils. The family Rosaceae was identified with OTU Sanguisorbinae, which includes Patagonian genus *Acaena*, found in medium abundances in both the Van Geel et al. (2022) coprolite and the McCulloch et al. (2021) core, but once again not in Moore’s macrofossil work. Family Poaceae was identified across four OTUs: Hordeinae, Tricitinae, Poeae Chloroplast group 1 and group 2. Of these OTUs Triticinae is not local to Patagonia, and while Chloroplast group 1 contains four local genera (Table S4), it also contains oats. However while these suggest possible reference bias attraction to commercial crop species, Hordeineae and Poeae Chloroplast group 1 contain eight local genera between them. Further, Poaceae remains are very abundant in the coprolites both in pollen and in macrofossil form, leading to the understanding that Mylodon were grazers (*e.g.*, Nordenskjöld, 1900; Moore, 1978; Markgraf, 1985). Additionally, they are the dominant pollen taxon in the environmental record for the timeline of the current dataset (McCulloch et al., 2021). Considering their dominance in both the environment and coprolites themselves, it is surprising that so little grass DNA was recovered. It is possible that with more sequencing and more local reference genomes the relative amount of Poaceae may improve.

The final two families identified, deserving mention as the OTUs match local taxa, are Chenopodiaceae (=Amaranthaceae) and Apiaceae (genus *Azorella*). All Chenopodiaceae genera in the plant list are in the Patagonian steppe, and this family had low abundances in pollen and macrofossils of coprolites (Moore, 1978; Van Geel et al., 2022) and the sediment core (McCulloch et al., 2021). *Azorella*, though only identified from a single hit, is an interesting identification as it is in high abundances in the pollen of the Van Geel et al. (2022) coprolite and low abundances in the older part of the sedimentary record (McCulloch et al., 2021).

There are a few marked absences in this study of plant families recorded as abundant in morphological works. The family Ericaceae, notably the genus *Empetrum*, is the most abundant taxa in the pollen of van Geel’s coprolites and in the younger coprolites studied by Markgraf (Markgraf, 1985; Van Geel et al., 2022). *Empetrum* is also identified from seeds (Moore, 1978), and the corresponding pollen records in the sediment core of Cerro Benitez (McCulloch et al., 2021). Asteraceae, the most abundant plant family in the Markgraf (1985) coprolites, was also absent, though only found in medium abundance in Van Geel et al. (2022), and under 10% around 15,000 cal yr BP from pollen (McCulloch et al., 2021). The final notable family absence is Cyperaceae, identified in low abundances by Markgraf (1985), found as seeds (Moore, 1978), and in very low abundances in the 16,300–15,000 BP sequence in the sediment core (McCulloch et al., 2021). Our limited dataset precludes a definitive explanation for the observed absences but could include poor taxa-specific DNA preservation, stochasticity due to the limited numbers of reads sequenced, or genuine absence. Whilst more DNA data would be needed to make strong ecological inference about the diet and environment of *M. darwinii*, we find that the vast majority of OTUs obtained through the BLAST-PIA pipeline are consistent with the Patagonian Steppe environment as previously identified for the chronology of our samples, which is encouraging for further palaeogenetic work.

Incomplete genomic reference databases are a challenge for any metagenomic analysis. The regional absence of high quality local taxa is problematic and further difficulties arise when trying to identify families whose closest relatives are commercial crops: in particular for grasses, which have twenty Patagonian genera but would match to the well-represented genomes of wheat, oats and rice. The method applied here, nuclear genome data combined with organelle sequences in the nucleotide database, as well as a local comparison database, allowed further identifications such as *Azorella* and *Stellaria* despite the conservative PIA algorithm and our small dataset. The development of a reference Patagonian flora database, such as PhyloNorway in the Arctic (Alsos et al., 2020), would be a great asset to future genetic and genomic investigations in this region.

## Conclusions

This exploration of DNA in *M. darwinii* coprolites and soil samples from Cueva del Milodón revealed their potential for recovering local and endogenous signals as well as radiocarbon measurements when adapted laboratory and analytical methods are used. Although the amount of data and read lengths were influenced by the extraction method, it seems the choice of extraction method will not bias the type of DNA recovered. Out of the three extraction methods tested we recommend the PowerSoil kit as the best performing protocol. This could therefore be a useful starting point for DNA recovery from mammalian coprolites, However it must be noted that there is much variation in both molecular preservation and chemical inhibition between localities, so the depositional environment of the coprolites should be considered as this may raise additional considerations regarding extraction methods.

## Supplemental Information

10.7717/peerj.21009/supp-1 Supplemental Information 1 Supplementary Methods Detailed methods for this study.

10.7717/peerj.21009/supp-2 Supplemental Information 2 Supplemental Figures Supplementary Figures 1 to 4, with legends enclosed.

10.7717/peerj.21009/supp-3 Supplemental Information 3 Radiocarbon date measurements and calibrations for specimens in this study The radiocarbon measurement and calibrations for the specimens of coprolites and coprolite fragments in this study. Columns include metadata of samples, radiocarbon measurements from ORAU, Calibration curve and notes, and calibrated ages means and 2sigma range in BCE and PB format.

10.7717/peerj.21009/supp-4 Supplemental Information 4 Extract and Library concentrations, and TapeStation regions examined Details on the libraries generated in this studies with regard to DNA yield. It has three sheets: 1) a ReadMe sheet describing sections and columns in the next two sheets; 2) Concentration_Statistics sheet, representing extract and library concentration statistics, both raw and scaled per mg sample used; 3) TapeStation_Regions, a sheet honing in on key regions of the TapeStation traces representing either very short reads or the general sample region of the libraries generated in this study.

10.7717/peerj.21009/supp-5 Supplemental Information 5 Sequencing and Mapping statistics for the libraries generated in the study Statistics on sequencing results, as well as mapping to the nuclear genome of two-toed sloth *Choloepus didactylus* (closest genetic relative to *Mylodon darwinii*) and to the mitochondrial genome of *M. darwinii*. It has two sheets: a ReadMe explaining all columns, and then MappingStats laying out summary metadata, extraction, library, sequencing and alignment to sloth statistics for each sample in this study.

10.7717/peerj.21009/supp-6 Supplemental Information 6 List of Patagonian vascular plant species and their ecological associations derived from Pisano (1977), and overlap with identified operational taxonomic units (OTUs) in this study Firstly sheet ReadMe describes the next two sheet headers in detail. Secondly, sheet BLAST_PIA_OTUs then shows a per-sample breakdown of read counts for all identified plant groups, whether they are found in the Patagonian plant list, and compare the resulting taxa with previous plant research in similar material. Finally, sheet Pisano1977_Moore1978list reports the taxonomy for all listed entries of vascular plants, as well as their associations in the plant communities found in Chilean Patagonia between 52 ° S and 56 ° S as well as in the direct surroundings of Cueva del Milodón.

## Institutional Abbreviations

NHMUK: Natural History Museum, London, United Kingdom
ORAU: Oxford Radiocarbon Accelerator Unit, Oxford, United Kingdom
DAB: Daicel Arbor Biosciences, Ann Arbor, United States.

## Additional Information and Declarations

### Data Availability

The following information was supplied regarding data availability: The raw data is available at Figshare: Zicos, Maria (2026). Mylodon darwinii coprolite raw reads. figshare. Dataset. https://doi.org/10.6084/m9.figshare.29635013.v1
The raw sequence data are available at GenBank: PRJNA1295883.
