# Supplementary material for: Megaherbivore coprolite DNA: yields and comparison of three ancient DNA extraction protocols on coprolites of giant ground sloth Mylodon darwinii
Source: PeerJ. 2026 Jun 12;14:e21009. doi: 10.7717/peerj.21009 (PMC13267792; doi:10.7717/peerj.21009)
Supplement: Supplemental Information 1 — Detailed methods for this study. [file peerj-14-21009-s001.docx]

Supplementary methods for: Megaherbivore coprolite DNA: yields and comparison of three ancient DNA extraction protocols on coprolites of giant ground sloth *Mylodon darwinii*.

Maria H. Zicos^1,2^, Ian Barnes^1^, Laurent A. F. Frantz^2,3^, Selina Brace^1^

^1^ Science Department, Natural History Museum, London, United Kingdom

^2^ School of Biological and Behavioural Sciences, Queen Mary University of London, London, United Kingdom

^3^ Faculty of Veterinary Medicine, Ludwig-Maximilians-Universität, Munich, Germany.

Institutional Abbreviations

NHMUK: Natural History Museum, London, United Kingdom

QMUL: Queen Mary University of London, London, United Kingdom

IdlP: Instituto de la Patagonia, Punta Arenas, Chile

ORAU: Oxford Radiocarbon Accelerator Unit, Oxford, United Kingdom

DAB: Daicel Arbor Biosciences, Ann Arbor, United States

Samples

Seven specimens from the NHMUK fossil mammal collection were sampled for this study. Two specimens were complete coprolites NHMUK PV M102296 and M 102299 assigned to *M. darwinii* from the historical collection of Cueva del Milodón . This “historical collection” is composed of Cueva del Milodón specimens acquired by the NHMUK (at the time British Museum, Natural History) from excavations led from the late 1890’s into the 1910’s. Modern archaeological/palaeontological methodologies were not yet applied, so these specimens lack information about location within the cave, depth and context.

The remaining five specimens were selected from soil specimens collected in an excavation led by Dr Earl C. Saxon from Durham University in early 1976, funded by the IdlP (henceforth the Saxon Excavation). The NHMUK’s portion of this collection includes owl pellets, small mammal bones and soil from Cueva del Milodón. Saxon’s excavation scheme included identifying main layers of the stratigraphy in three trenches excavated at the site (trenches 2a/7, 3, 5 and a midden named A; see Fig. 1A), as well as collecting in spits of 5 cm depth (Saxon 1976, 1979). Regrettably, only one stratigraphic profile exists in publication for trench 2 (Fig. 2B in Saxon 1979) but there is a list of layers - and spits within - in related publications (Table 1 in Saxon 1976 and Table 1 in Moore 1978), and in written notes in the NHMUK Palaeontology collection. The archaeological and large mammal finds were returned to the IdlP, and small mammals and soil brought to the UK and US for further study.

The soil specimens in the Saxon Collection from the 1976 excavation of Cueva del Milodón were collected into bags representing 5cm spits within layers of the cave. The five samples for this study were taken from layer 3 of trench 5, representing the known layer of compacted *Mylodon* dung which could reach approximately 1m in depth in sections of the cave (Hauthal et al. 1899; Nordenskjold 1900). This layer was divided into 15 sections by the Saxon Excavation, labelled 5.3D1 to 5.3A.15 (Fig. 1B). The five samples for this study were selected from subsections of layer 3 as follows: the top (5.3D.1), bottom (5.3A.15) and middle (5.3C.6), as well as two others between the middle and extremities of the dung layer (5.3D.3 and 5.3B.9).


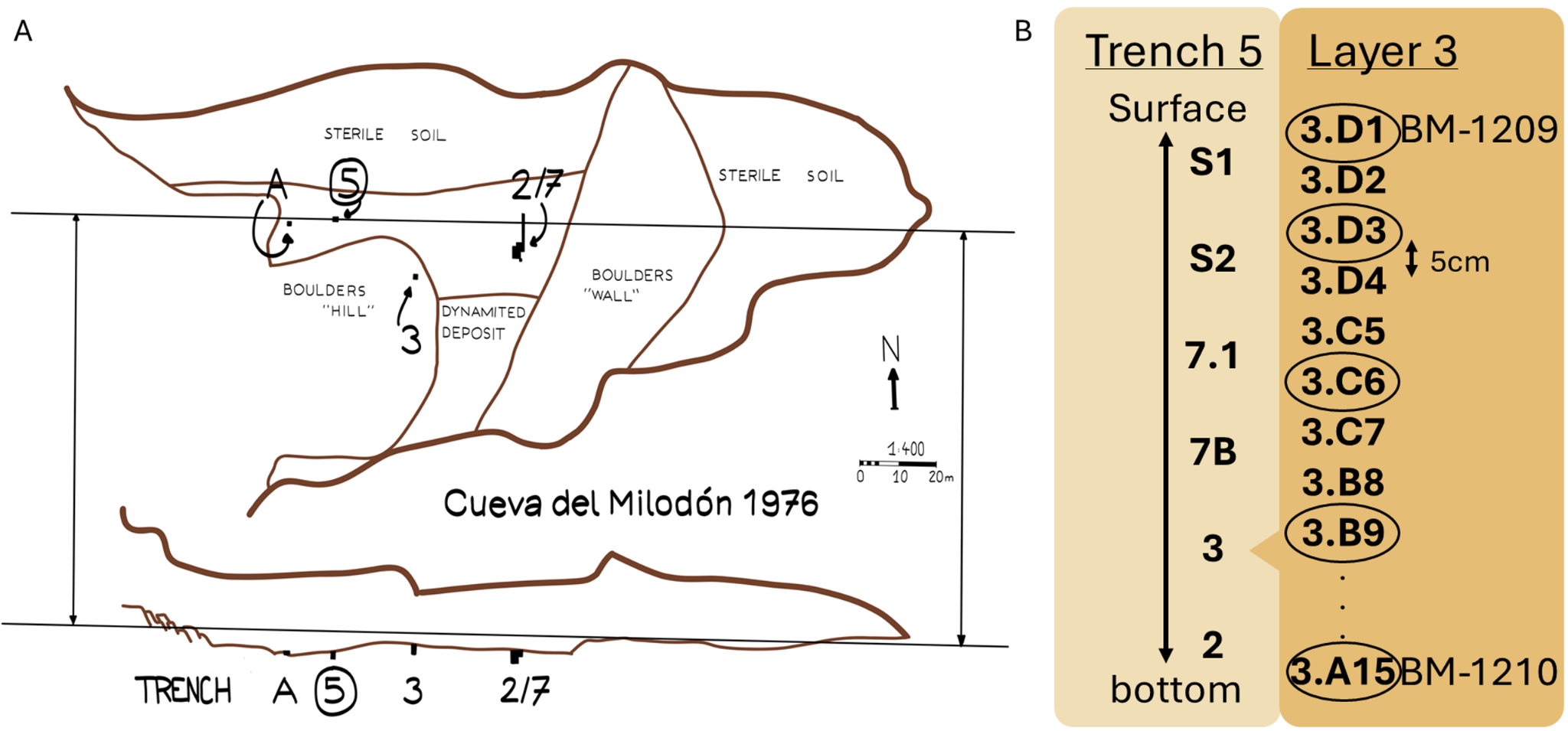


Fig 1. A. Location of the trenches excavated in 1976 in Cueva del Milodón. B. layers of trench 5, with layer 3 (the compacted dung layer) in detail. Selected samples are shown circled, and top and bottom conventional radiocarbon lab numbers are shown.

Sampling and Radiocarbon Dating

Sampling was conducted in the NHMUK ancient DNA laboratory, in a sampling hood with UV lights. The sampling hood was UV-radiated before and after every sampling session, as well as cleaned with bleach before and after sampling each coprolite specimen. The specimens were sampled using sterile scalpels, which were cleaned with DNA-away and UV-radiated for at least 10 minutes before and after each use. For the two historical coprolites, care was taken not to sample from the exterior of the bolus. In the interest of limiting the destruction on the well-preserved specimens of coprolites in the historical collection, these were not bisected and sampled from the centre, as recommended in best practice (Wood and Wilmshurst2016). Instead, a small section of the outermost part of the coprolites were removed prior to sampling, and the sample was taken in the “clean”, previously unexposed, area. The Saxon collection samples were taken as such: one large clump was selected from the specimen bag, bisected, and the centre portion sampled following Wood and Wilmshurst (2016). All coprolite specimens were subsampled three times for sample weights required by the different extraction methods.

Sampling for radiocarbon dating was performed similarly with a sample weight aim of 1g and sent to the Oxford Radiocarbon Accelerometer Unit for Accelerator Mass Spectrometry radiocarbon dating (Brock et al. 2010). The radiocarbon measurements were calibrated in Oxcal online v.4.4.5 (Bronk Ramsay 2021) using SHCal20 (Hogg et al. 2020).

Extractions

The laboratory procedures for DNA extraction, and library preparation prior to amplification, were carried out in a dedicated and purpose-built ancient DNA laboratory at the NHMUK.

Extractions and library preparations were carried out in a dedicated hood (different from the sampling hood). For each sampled specimen, extractions were carried out using three protocols on subsamples, chosen to address concerns of inhibition in the samples: firstly, a slightly modified version of an established protocol specialised for the recovery of small fragments in the field of ancient DNA (Dabney et al. 2013; modified as in Brace et al. 2019), originally developed for DNA extractions from bone and modified for extractions from tissue (from now on referred to as Dabney Tissue protocol; modifications described below), which usually requires up to 50 mg of sample. The second protocol was the Qiagen DNeasy Plant mini kit (purchased 2018), and the third was the Qiagen DNeasy PowerSoil kit (purchased 2018), designed to maximise DNA recovery from plant material and microorganisms in soil, respectively. The Qiagen DNeasy Plant mini kit, which calls for 20-100 mg of starting material, was selected based on its use in the aDNA literature (Lendvay et al. 2018; Gould et al. 2010; Kistler and Shapiro 2011; Li et al. 2016; Suyama et al. 2008; Costa and Roberts 2014) with the aim to recover plant DNA from the coprolites of the herbivore depositor. The PowerSoil kit, requiring 250 mg of sample, was selected for its utility in removing DNA inhibitors often found in soil and faecal samples, making it widely used in the aDNA soil and faecal research literature (Seersholm et al. 2016; Cano et al. 2014; Soe et al. 2015; Wood et al. 2012; Boast et al. 2018).

The following modifications were carried out on the extraction protocols: a) in the Dabney Tissue protocol the lysis buffer was Qiagen ATL buffer (180 μL) with proteinase K (20 μL, 10mg/μL). Samples were incubated for 24 hours at 56°C under constant rotation at 48 rpm. The remainder of the protocol is identical to Brace et al. (2019). b) in the DNeasy Plant mini kit extractions, the incubation period was increased to two hours with rotation at 48 rpm following recommendations in the literature for extended lysis incubation period (Wales et al. 2019). All optional cold incubation precipitation stages in the kit manual were followed. c) In the PowerSoil kit, after rehydrating the sample through addition of the bead solution and lysis buffer for the cell lysis stage, rather than vortexing the samples for ten minutes in a vortex adapter, they were vortexed at maximum speed for one minute, then incubated at room temperature for 20 min at 80 rpm. Extract concentrations were quantified with a qubit HS DNA assay on a qubit 2.0.

Libraries and Sequencing

Libraries were prepared from 30μl of extract, using a double-stranded library protocol adapted from Meyer and Kircher (2010). This protocol includes a blunt-end repair step, ligation and fill in of adapters. Indexing PCR was carried out by double-indexing each sample with unique barcodes (3 replicate PCR reactions conducted in the NHM molecular laboratory). All libraries were amplified for 20 cycles and eluted to 50 μL. Libraries were quantified on an Agilent TapeStation 2200 using a D1000 tape and reagents.

Shotgun libraries were sequenced on paired end 75bp runs on mid-output kits using the NextSeq 500 at the NHM sequencing facility. The libraries from the Dabney Tissue protocol were sequenced on a separate run to the PowerSoil and Plant kit libraries. Additional to shotgun sequencing, seven aliquots from the PowerSoil-extracted libraries (and one Dabney-extracted library for which the corresponding PowerSoil sample had low DNA concentration) were sent to DAB for hybridisation capture of the mitochondrial genome. The capture baits were based on the full mitochondrial genome of *Mylodon darwinii*, the only ground sloth known at the site, as generated by Delsuc et al. (2018; Genbank accession MF061314.1 or NC_037941.1). The probes were generated as 80-mer with 4x tiling. Capture was performed in 6 double reactions following protocol described by Delsuc and colleagues (2019). These were then sequenced as part of a Novaseq S4 lane on a paired end 150 bp sequencing run.

Data Preprocessing

Raw reads in fastq format for each sample were quality-checked using FastQC (http://www.bioinformatics.babraham.ac.uk/projects/fastqc) before any manipulation. Adapters were removed using AdapterRemoval v2.2.2 (Lindgreen, 2016), with additional settings to exclude sequences shorter than a minimum read threshold of 25 base pairs and bases assigned a Phred score lower than 30 during the sequencing. Read merging was conducted on reads with the default minimum overlap of 11bp (resulting in a maximum collapsed read length of 141bp). Duplicates were removed from the collapsed reads using Prinseq (Schmieder and Edwards 2011). The collapsed and de-duplicated reads were used in the alignment pipelines to sloth as well as the taxonomic classification pipeline.

Alignments to Sloth

Reads from the shotgun libraries and the captured libraries were mapped against reference genomes using BWA (v 0.7.17-r1188; Li and Durbin, 2009). No nuclear reference genomes currently exist for extinct sloths. Consequently, the closest living relative of *Mylodon*, the southern two-toed sloth *Choloepus didactylus*, was used as a reference (Vertebrate Genome Project https://vgp.github.io/genomeark/Choloepus_didactylus/ ; Genbank assembly accession GCA_015220235.1). The mitochondrial genome of *Mylodon darwinii* (Delsuc et al. 2018; Genbank accession MF061314.1 or NC_037941.1) was used as a reference for mitochondrial analysis. Mapping was performed with algorithm aln, with seed disabling (-l 1024) and mapping parameters -n 0.01 (which allows for substitutions relative to the reference) and -o 2 (allowing up to two gaps), following Pečnerová and colleagues (Pečnerová et al. 2017).

After aligning, the libraries were converted to sam format using bwa function samse, and to bam format using SAMTOOLS (v 1.12; Li et al., 2009). Samtools was then used to sort the libraries (sort), remove duplicates (rmdup), and filter the bam files based on mapping quality. For mitochondrial genome mapping, the quality threshold was set at 30, as usually done when the reference genome and study species are the same.

Endogenous content was calculated as the proportion of reads mapping to the reference nuclear genome after removal of duplicates and quality filtering (mapping quality 20 as the reference and samples are different species). Coverage depth was determined using Qualimap2 (v 2.2.2a; Okonechnikov, Conesa and García-Alcalde, 2016). Ancient DNA authentication was performed by checking the mapped reads for damage patterns typical of ancient DNA using MapDamage (v2; Jónsson et al. 2013), and the bam files were rescaled to set Phred Scores to 0 for bases likely to be deamination damage (option --rescale).

Taxonomic classification

Exploration of the taxonomic diversity of the samples was performed using BLASTn and PIA.

Fastq files with adapters removed were run through BLAST (Camacho et al. 2009) against the NCBI nucleotide database (downloaded 19^th^ January 2019), and the resulting outputs were run through PIA (Cribdon et al. 2020) with default settings for identifying Phylogenetic intersection. The outputs for each sample were merged using script Collate_Summary_Basics.pl from PIA_accessories (Cribdon et al. 2020). A custom script was used to keep only the Taxids and identified reads per sample in the output of the collate_summary_basics.pl script, generating an OTU table in a suitable format for import into to R and R studio (R 4.2.3,  R Core Team 2023; R studio RStudio 2023.03.0+386 "Cherry Blossom”, Posit team 2023) for analysis using packages Phyloseq (v1.40.0; McMurdie Holmes 2013 ) and genefilter (version 1.78.0; Gentleman et al. 2022), with visualisations using ggplot2 (Tidiverse package suite, Wickham et al. 2016, 2019).

Package Taxonomizr (v 0.10.2; Sherrill-Mix 2023) in R was used to generate a taxonomy table from the Taxids in the PIA results, displaying the same taxonomic levels as those in the kraken biom files for comparison of the methods. Finally, these Taxonomy and OTU tables were combined with sample metadata into one Phyloseq object using function phyloseq.

The ecological properties of the sequenced metagenomes were described through alpha diversity metrics of richness and evenness (species richness, Shannon index and Simpson Index) performed in package phyloseq (Shannon and Weaver 1949; Simpson 1949; McMurdie and Holmes 2013), which were compared through Kruskal-Wallis tests (Kruskal & Wallis 1952). If there was evidence of significant differences, the variable was further examined for the effect size of that difference (Tomczak and Tomczak 2014) and  which pairwise state of the covariates were different with Wilcoxon rank-sum tests corrected for multiple comparison (Wilcoxon 1945; Dunn 1961; Benjamini and Hochberg 1995).

Taxonomic classification verification

Two main publications were selected for creating a list of plant species existing near Cueva del Milodón. To begin with, a list was collected of plant species in the four main types of plant communities of Chilean Patagonia between latitudes 52° and 56° S, as described by Pisano (1977). This range was selected to encompass a wider variety of plants than those in the direct vicinity of Cueva del Milodón, as the paleoenvironment of the cave changed through time and the plant assemblage near the site could have been more similar to those not currently found there. This was supplemented by Moore’s (1978) list of plants around Cueva del Milodón.

The taxa in the dataset were manually checked against modern taxonomy using The Plant list (<http://www.theplantlist.org/>) and GBIF (https://www.gbif.org/). Additional to checking verified names for these species, higher taxonomic classification was collected, so that this dataset could be compared with the results of the taxonomic classification by the BLAST-PIA pipeline. The verified taxonomy was replaced in the list. If a species name was unverified, the genus would be kept and the species listed as “spp.” in the corrected list. Once corrected genus and species names were incorporated in the list, the higher taxonomy was added to the original descriptions of Patagonian plant assemblages. For genera that were not found in online resources, an effort was made to find the higher taxonomic levels of that species based in context in the publication. If this information was found, the record would be included in the plant list, listed as “undet” in all levels below the lowest taxonomic level found. In one instance no information was found at all (genus *Australophozia*) and so this record was deleted from the plant list.

Taxonomic identifications in the samples were compared against this list to determine whether the taxonomic assignments were likely to represent local flora. This was mainly done using the taxonomy table in R, but to better understand the identification and whether they represent a local signal, further information was leveraged from the taxid number.

Once taxonomic classifications were produced, community composition was summarised in two dimensions from sample data normalised for sequencing depth through non-metric multidimensional scaling (NMDS; Bray-curtis Distance metric, vegan package in R; Bray and Curtis 1957; Kruskal 1964; Oksanen et al. 2024).

Properties of the generated data

To study the characteristics of the different extractions on our extracts and libraries, the following metrics were compared with non-parametric difference testing: 1) Extract concentration (representing DNA recovered at extraction), 2) library concentration after amplification (representing realised DNA yield), 3) sequenced read lengths, 4) library complexity (representing proportions of unique molecules in the sequenced libraries), 5) proportion of identified reads, and finally 6) proportion of identified plant reads. Complexity of sequenced libraries was computed along with DNA concentrations as a separate measure of library quality to distinguish between abundance of DNA (concentration) and amount of information in the sequenced DNA (complexity), as a high concentration does not guarantee high complexity. The comparisons were conducted with non-parametric Kruskal-Wallis tests when the values were one datum per library (all metrics above except read length), and with ANOVA (Fisher 1934) for the read length analysis (as the sample size was all reads in the collapsed fastq files for the whole experiment).

For read lengths, the difference tests were first conducted on the whole dataset of read lengths for collapsed reads (n=58.34 million reads excluding controls), reconstructed from AdapterRemoval run summaries. They were also conducted on subsets of randomly sampled reads in the libraries to present equal sample sizes. The first subsetted read length dataset was a random selection of 500 reads per library where such numbers were available for all three replicates of the specimen, to test for difference in read lengths due to the extraction method. The second subset was a selection of 1000 reads per sample type (n=2000) to have even sample sizes to test for read length differences between true coprolites and samples from the Saxon trench dung layer. The third subset was a selection of 500 reads per specimen (n=3500 reads total) to even out sample sizes for testing for differences between sample age.

Additionally, in order to examine the recovery of short fragments, non-parametric difference testing was conducted on average fragment length, molarity and percentage of the sample contained in the fragment size regions of 155-180bp in the library TapeStation traces. As adapter dimer peaks generally span the 140-150bp region, this region represents short insert sizes, up to about 35-40 bp (comparison with the “full library” region of 155-400bp in SI Fig. 3).

For difference testing of the library complexity, libraries which failed to amplify (n=1/7 in Dabney libraries, n=1/7 in Plant kit libraries) or had an extremely low concentration relative to the rest of the replicates (n=1/7 in soil kit libraries) were excluded from the comparison. In an attempt to control for sequencing effort, read complexity was calculated both as the raw duplication rate (percentage of reads in the collapsed fastqs that were not unique molecules) and a normalised duplication rate, per million sequenced reads (raw duplication rate divided by million sequenced reads).

Further, the normalised NMDS community composition was tested for differences in extraction protocols, as sample type and Radiocarbon age of the specimens were. This exploration was carried out through Mantel tests and ANOSIM tests respectively (Legendre and Legendre 2012; Clarke 1993; Warton, Wright, and Wang, 2012).

References

Benjamini Y, Hochberg Y. 1995. Controlling the False Discovery Rate: A Practical and Powerful Approach to Multiple Testing. *Journal of the Royal Statistical Society Series B: Statistical Methodology* 57:289–300. DOI: 10.1111/j.2517-6161.1995.tb02031.x.

Boast AP, Weyrich LS, Wood JR, Metcalf JL, Knight R, Cooper A. 2018. Coprolites reveal ecological interactions lost with the extinction of New Zealand birds. *Proceedings of the National Academy of Sciences* 115:1546–1551. DOI: 10.1073/pnas.1712337115.

Brace S, Diekmann Y, Booth TJ, van Dorp L, Faltyskova Z, Rohland N, Mallick S, Olalde I, Ferry M, Michel M, Oppenheimer J, Broomandkhoshbacht N, Stewardson K, Martiniano R, Walsh S, Kayser M, Charlton S, Hellenthal G, Armit I, Schulting R, Craig OE, Sheridan A, Parker Pearson M, Stringer C, Reich D, Thomas MG, Barnes I. 2019. Ancient genomes indicate population replacement in Early Neolithic Britain. *Nature Ecology & Evolution* 3:765–771. DOI: 10.1038/s41559-019-0871-9.

Bray JR, Curtis JT. 1957. An Ordination of the Upland Forest Communities of Southern Wisconsin. *Ecological Monographs* 27:325–349. DOI: 10.2307/1942268.

Brock F, Higham T, Ditchfield P, Ramsey CB. 2010. Current Pretreatment Methods for AMS Radiocarbon Dating at the Oxford Radiocarbon Accelerator Unit (Orau). *Radiocarbon* 52:103–112. DOI: 10.1017/S0033822200045069.

Bronk Ramsey C. 2023. OxCal v4.4.5.

Camacho C, Coulouris G, Avagyan V, Ma N, Papadopoulos J, Bealer K, Madden TL. 2009. BLAST+: architecture and applications. *BMC Bioinformatics* 10:421.

Cano RJ, Rivera-Perez J, Toranzos GA, Santiago-Rodriguez TM, Narganes-Storde YM, Chanlatte-Baik L, García-Roldán E, Bunkley-Williams L, Massey SE. 2014. Paleomicrobiology: Revealing fecal microbiomes of ancient indigenous cultures. *PLoS ONE* 9. DOI: 10.1371/journal.pone.0106833.

Clarke KR. 1993. Non-parametric multivariate analyses of changes in community structure. *Austral Ecology* 18:117–143. DOI: 10.1111/j.1442-9993.1993.tb00438.x.

Costa CM, Roberts RP. 2014. Techniques for improving the quality and quantity of DNA. *Phytoneuron* 48:1–8.

Cribdon B, Ware R, Smith O, Gaffney V, Allaby RG. 2020. PIA: More Accurate Taxonomic Assignment of Metagenomic Data Demonstrated on sedaDNA From the North Sea. *Frontiers in Ecology and Evolution* 8:1–12. DOI: 10.3389/fevo.2020.00084.

Dabney J, Knapp M, Glocke I, Gansauge M-T, Weihmann A, Nickel B, Valdiosera C, Garcia N, Paabo S, Arsuaga J-L, Meyer M. 2013. Complete mitochondrial genome sequence of a Middle Pleistocene cave bear reconstructed from ultrashort DNA fragments. *Proceedings of the National Academy of Sciences* 110:15758–15763. DOI: 10.1073/pnas.1314445110.

Delsuc F, Kuch M, Gibb GC, Hughes J, Szpak P, Southon J, Enk J, Duggan AT, Poinar HN. 2018. Resolving the phylogenetic position of Darwin’s extinct ground sloth ( Mylodon darwinii ) using mitogenomic and nuclear exon data. *Proceedings of the Royal Society B: Biological Sciences* 285:20180214. DOI: 10.1098/rspb.2018.0214.

Delsuc F, Kuch M, Gibb GC, Karpinski E, Hackenberger D, Szpak P, Martínez JG, Mead JI, McDonald HG, MacPhee RDE, Billet G, Hautier L, Poinar HN. 2019. Ancient Mitogenomes Reveal the Evolutionary History and Biogeography of Sloths. *Current Biology* 29:2031-2042.e6. DOI: 10.1016/j.cub.2019.05.043.

Dunn OJ. 1961. Multiple Comparisons Among Means. *Journal of the American Statistical Association* 56:52–64. DOI: 10.1080/01621459.1961.10482090.

Fisher RA. 1934. *Statistical methods for research workers.* Oliver and Boyd, Edinburgh and London.

Gentleman R, Carey VJ, Huber W, Hahne F. 2022. genefilter: genefilter: methods for filtering genes from high-throughput experiments.

Gould BA, León B, Buffen AM, Thompson LG. 2010. Evidence of a high-andean, mid-holocene plant community: An ancient DNA analysis of glacially preserved remains. *American Journal of Botany* 97:1579–1584. DOI: 10.3732/ajb.1000058.

Hauthal R, Roth S, Lehmann-Nitsche R. 1899. Reseña de los hallazgos en las cavernas de Última Esperanza (Patagonia Austral). *Revista del Museo de la* 9:411–420.

Hogg AG, Heaton TJ, Hua Q, Palmer JG, Turney CSM, Southon J, Bayliss A, Blackwell PG, Boswijk G, Bronk Ramsey C, Pearson C, Petchey F, Reimer P, Reimer R, Wacker L. 2020. SHCal20 Southern Hemisphere Calibration, 0-55,000 Years cal BP. *Radiocarbon* 62:759–778. DOI: 10.1017/RDC.2020.59.

Jónsson H, Ginolhac A, Schubert M, Johnson PLF, Orlando L. 2013. mapDamage2.0: fast approximate Bayesian estimates of ancient DNA damage parameters. *Bioinformatics* 29:1682–1684. DOI: 10.1093/bioinformatics/btt193.

Kistler L, Shapiro B. 2011. Ancient DNA confirms a local origin of domesticated chenopod in eastern North America. *Journal of Archaeological Science* 38:3549–3554. DOI: 10.1016/j.jas.2011.08.023.

Kruskal JB. 1964. Nonmetric multidimensional scaling: A numerical method. *Psychometrika* 29:115–129. DOI: 10.1007/BF02289694.

Kruskal WH, Wallis WA. 1952. Use of ranks in one-criterion variance analysis. *Journal of the American statistical Association* 47:583–621.

Legendre P, Legendre L. 2012. *Numerical Ecology*. Elsevier.

Lendvay B, Hartmann M, Brodbeck S, Nievergelt D, Reinig F, Zoller S, Parducci L, Gugerli F, Büntgen U, Sperisen C. 2018. Improved recovery of ancient DNA from subfossil wood - application to the world’s oldest Late Glacial pine forest. *New Phytologist* 217:1737–1748. DOI: 10.1111/nph.14935.

Li C, Dong Y, Liu M, Lu P, Li W, Wang Y, Cui X, Zhou H, Xu Y. 2016. Ancient DNA analysis of Panicum miliaceum (broomcorn millet) from a Bronze Age cemetery in Xinjiang, China. *Vegetation History and Archaeobotany* 25:469–477. DOI: 10.1007/s00334-016-0561-3.

Li H, Durbin R. 2009. Fast and accurate short read alignment with Burrows-Wheeler transform. *Bioinformatics* 25:1754–1760. DOI: 10.1093/bioinformatics/btp324.

Li H, Handsaker B, Wysoker A, Fennell T, Ruan J, Homer N, Marth G, Abecasis G, Durbin R. 2009. The Sequence Alignment/Map format and SAMtools. *Bioinformatics* 25:2078–2079. DOI: 10.1093/bioinformatics/btp352.

McMurdie PJ, Holmes S. 2013. Phyloseq: An R Package for Reproducible Interactive Analysis and Graphics of Microbiome Census Data. *PLoS ONE* 8. DOI: 10.1371/journal.pone.0061217.

Meyer M, Kircher M. 2010. Illumina sequencing library preparation for highly multiplexed target capture and sequencing. *Cold Spring Harbor Protocols* 5. DOI: 10.1101/pdb.prot5448.

Moore DM. 1978. Post-glacial vegetation in the South Patagonian territory of the giant ground sloth, Mylodon. *Botanical Journal of the Linnean Society* 77:177–202. DOI: 10.1111/j.1095-8339.1978.tb01398.x.

Nordenskjöld E. 1900. Jakttagelser och fynd i Grottor vid Ultima Esperanza i sydvestra Patagonien. *Kongliga Svenska Vetenskaps-Akademiens Handlingar* 11:1–23.

Okonechnikov K, Conesa A, García-Alcalde F. 2016. Qualimap 2: advanced multi-sample quality control for high-throughput sequencing data. *Bioinformatics* 32:292–294. DOI: 10.1093/bioinformatics/btv566.

Oksanen J, Simpson GL, Blanchet FG, Kindt R, Legendre P, Minchin PR, O’Hara RB, Solymos P, Stevens MHH, Szoecs E, Wagner H, Barbour M, Bedward M, Bolker B, Borcard D, Carvalho G, Chirico M, De Caceres M, Durand S, Evangelista HBA, FitzJohn R, Friendly M, Furneaux B, Hannigan G, Hill MO, Lahti L, McGlinn D, Ouellette M-H, Ribeiro Cunha E, Smith T, Stier A, Ter Braak CJF, Weedon J. 2022. vegan: Community Ecology Package.

Pečnerová P, Palkopoulou E, Wheat CW, Skoglund P, Vartanyan S, Tikhonov A, Nikolskiy P, van der Plicht J, Díez-del-Molino D, Dalén L. 2017. Mitogenome evolution in the last surviving woolly mammoth population reveals neutral and functional consequences of small population size. *Evolution Letters* 1:292–303. DOI: 10.1002/evl3.33.

Pisano E. 1977. Fitogeografía de Fuego - Patagonia chilena. I.- Comunidades vegetales entre las latitudes 52 y 56^o^ S. *Anales del Instituto de la Patagonia* 8:121–250.

Posit team. 2023. RStudio: Integrated Development Environment for R.

R Core Team. 2023. R: A Language and Environment for Statistical Computing.

Saxon EC. 1976. La prehistoria de Fuego-Patagonia: Colonizacion de un Habitat Marginal. *Anales del Instituto de La Patagonia* 7:63–73.

Saxon EC. 1979. Natural prehistory: the archaeology of Fuego-Patagonian ecology. *Quaternaria* XXI:329–356.

Schmieder R, Edwards R. 2011. Quality control and preprocessing of metagenomic datasets. *Bioinformatics* 27:863–864. DOI: 10.1093/bioinformatics/btr026.

Schubert M, Lindgreen S, Orlando L. 2016. AdapterRemoval v2: Rapid adapter trimming, identification, and read merging. *BMC Research Notes* 9:1–7. DOI: 10.1186/s13104-016-1900-2.

Shannon CE, Weaver W. 1949. *The Mathematical Theory of Communication*. Urbana: University of Illinois Press.

Sherrill-Mix S. 2023. taxonomizr: Functions to Work with NCBI Accessions and Taxonomy.

Simpson EH. 1949. Measurement of Diversity. *Nature* 163:688–688. DOI: 10.1038/163688a0.

Søe MJ, Nejsum P, Fredensborg BL, Kapel CMO. 2015. DNA Typing of Ancient Parasite Eggs from Environmental Samples Identifies Human and Animal Worm Infections in Viking-Age Settlement. *Journal of Parasitology* 101:57–63. DOI: 10.1645/14-650.1.

Søe MJ, Nejsum P, Seersholm FV, Fredensborg BL, Habraken R, Haase K, Hald MM, Simonsen R, Højlund F, Blanke L, Merkyte I, Willerslev E, Kapel CMO. 2018. Ancient DNA from latrines in Northern Europe and the Middle East (500 BC–1700 AD) reveals past parasites and diet. *PLoS ONE* 13:1–17. DOI: 10.1371/journal.pone.0195481.

Suyama Y, Gunnarsson U, Parducci L. 2008. Analysis of short DNA fragments from Holocene peatmoss samples. *Holocene* 18:1003–1006. DOI: 10.1177/0959683608093540.

Tomczak MT, Tomczak E. 2014. The Need to Report Effect Size Estimates Revisited. An Overview of Some Recommended Measures of Effect Size. *Trends in SportSciences*.

Wales N, Akman M, Watson RHB, Sánchez Barreiro F, Smith BD, Gremillion KJ, Gilbert MTP, Blackman BK. 2019. Ancient <scp>DNA</scp> reveals the timing and persistence of organellar genetic bottlenecks over 3,000 years of sunflower domestication and improvement. *Evolutionary Applications* 12:38–53. DOI: 10.1111/eva.12594.

Warton DI, Wright ST, Wang Y. 2012. Distance-based multivariate analyses confound location and dispersion effects. *Methods in Ecology and Evolution* 3:89–101. DOI: 10.1111/j.2041-210X.2011.00127.x.

Wickham H. 2016. *ggplot2: Elegant Graphics for Data Analysis*. Springer-Verlag New York.

Wickham H, Averick M, Bryan J, Chang W, McGowan L, François R, Grolemund G, Hayes A, Henry L, Hester J, Kuhn M, Pedersen T, Miller E, Bache S, Müller K, Ooms J, Robinson D, Seidel D, Spinu V, Takahashi K, Vaughan D, Wilke C, Woo K, Yutani H. 2019. Welcome to the Tidyverse. *Journal of Open Source Software* 4:1686. DOI: 10.21105/joss.01686.

Wilcoxon F. 1945. Individual comparisons by ranking methods. *Biomedical Bulletin* 1:80–83.

Wood JR, Wilmshurst JM. 2016. A protocol for subsampling Late Quaternary coprolites for multi-proxy analysis. *Quaternary Science Reviews* 138:1–5. DOI: 10.1016/j.quascirev.2016.02.018.

Wood JR, Wilmshurst JM, Wagstaff SJ, Worthy TH, Rawlence NJ, Cooper A. 2012. High-resolution coproecology: Using coprolites to reconstruct the habits and habitats of New Zealand’s extinct upland Moa (Megalapteryx didinus). *PLoS ONE* 7. DOI: 10.1371/journal.pone.0040025.
