# Supplementary material for: Megaherbivore coprolite DNA: yields and comparison of three ancient DNA extraction protocols on coprolites of giant ground sloth Mylodon darwinii
Source: PeerJ. 2026 Jun 12;14:e21009. doi: 10.7717/peerj.21009 (PMC13267792; doi:10.7717/peerj.21009)
Supplement: Supplemental Information 2 — Supplementary Figures 1 to 4, with legends enclosed. [file peerj-14-21009-s002.docx]

Supplementary figures for: Megaherbivore coprolite DNA: yields and comparison of three ancient DNA extraction protocols on coprolites of giant ground sloth *Mylodon darwinii*.

Maria H. Zicos^1,2^, Ian Barnes^1^, Laurent A. F. Frantz^2,3^, Selina Brace^1^

^1^ Science Department, Natural History Museum, London, United Kingdom

^2^ School of Biological and Behavioural Sciences, Queen Mary University of London, London, United Kingdom

^3^ Faculty of Veterinary Medicine, Ludwig-Maximilians-Universität, Munich, Germany.


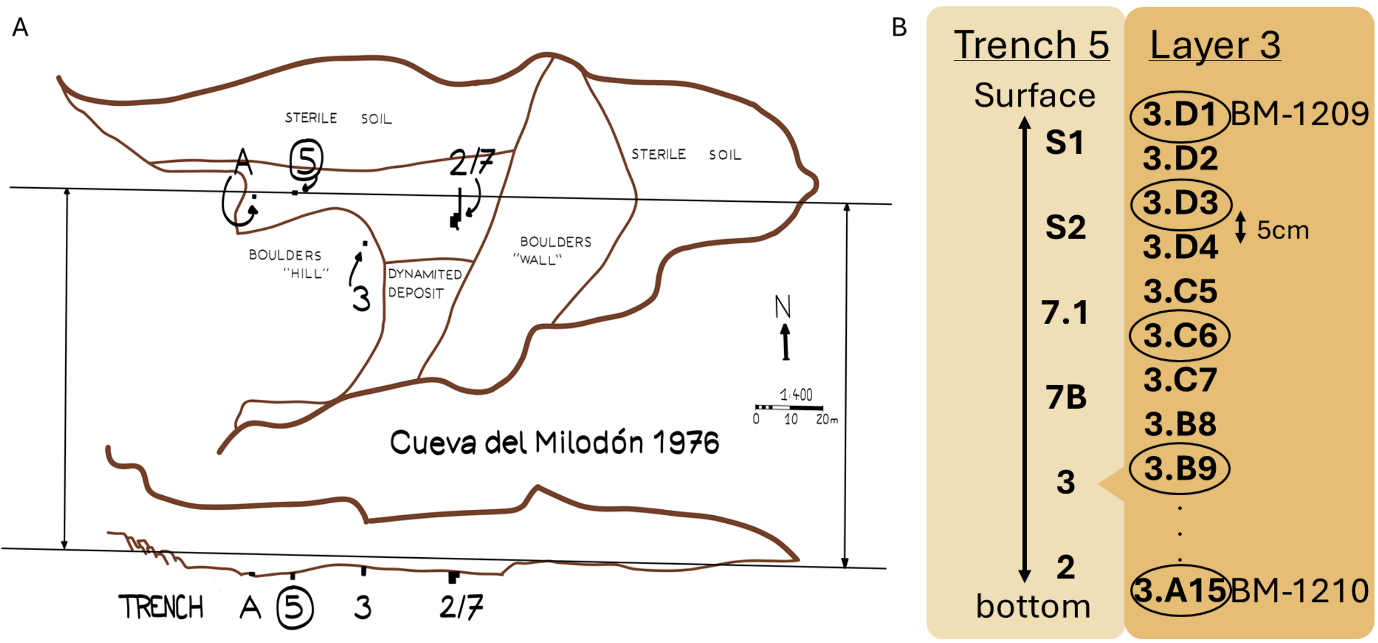


SI Figure 1: Excavation Schematics of Cueva del Milodón for trenches in the 1976 excavation led by Saxon (adapted from Fig 2a in Saxon 1979). A) map of Cueva del Milodón showing trenches excavated by Saxon in 1976 (adapted from Fig. 2a in Saxon 1979). Top = top down view of the cave showing outline and trench locations; bottom = transverse view of the cave. Trench 5, from which five samples in this study originate, is circled. B) Sequence of layers and sampled sections in trench 5, adapted from a written note by Saxon in the NHMUK collections. Each spit was sampled every 5 cm (Moore 1978) and bags of sediment (and/or sloth dung) were collected and kept in the UK with permission (Saxon 1978, handwritten note in NHMUK collection). Sections sampled for this study are shown circled. Laboratory numbers for existing conventional radiocarbon dates are provided.


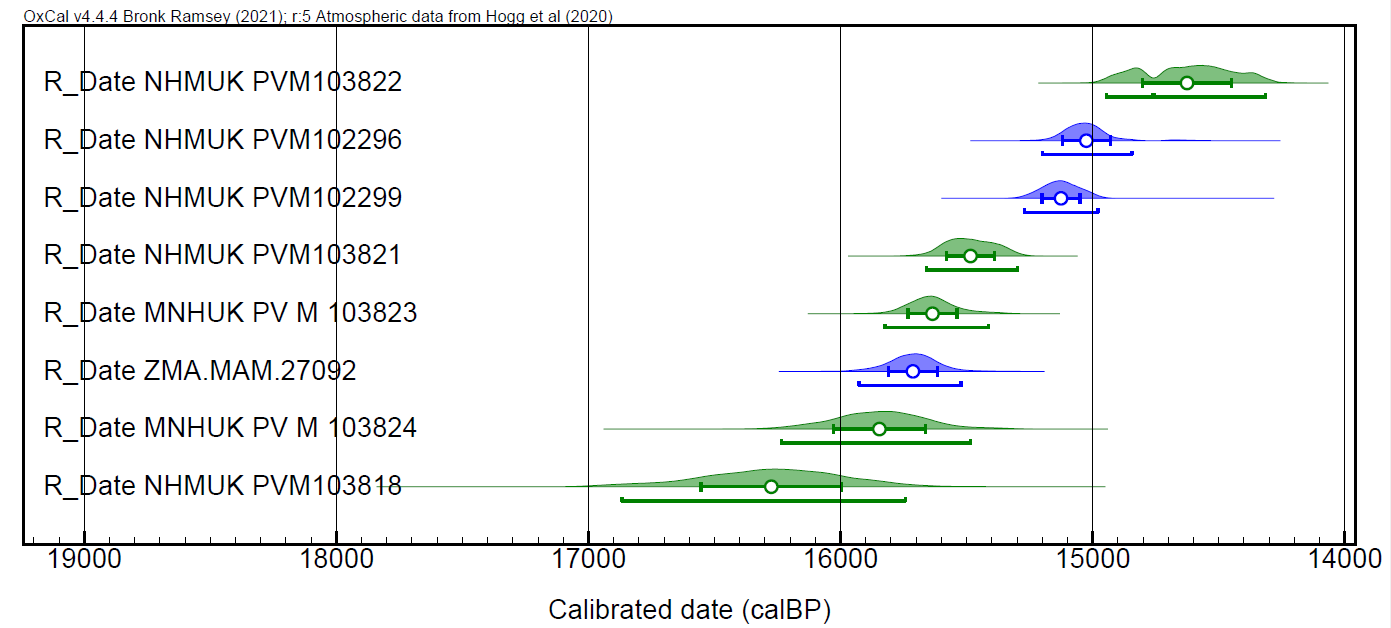


Supplementary Fig. 2. Calibrated AMS radiocarbon dates of coprolites from Cueva del Milodón. Mixed sediment and coprolite in green, full coprolites in blue. NHMUK specimens are from this study, and date GrM21,338 from specimen ZMA.MAM.27092 is from Van Geel et al. 2022. Raw radiocarbon measurements and calibrations are available in SI Table 1.


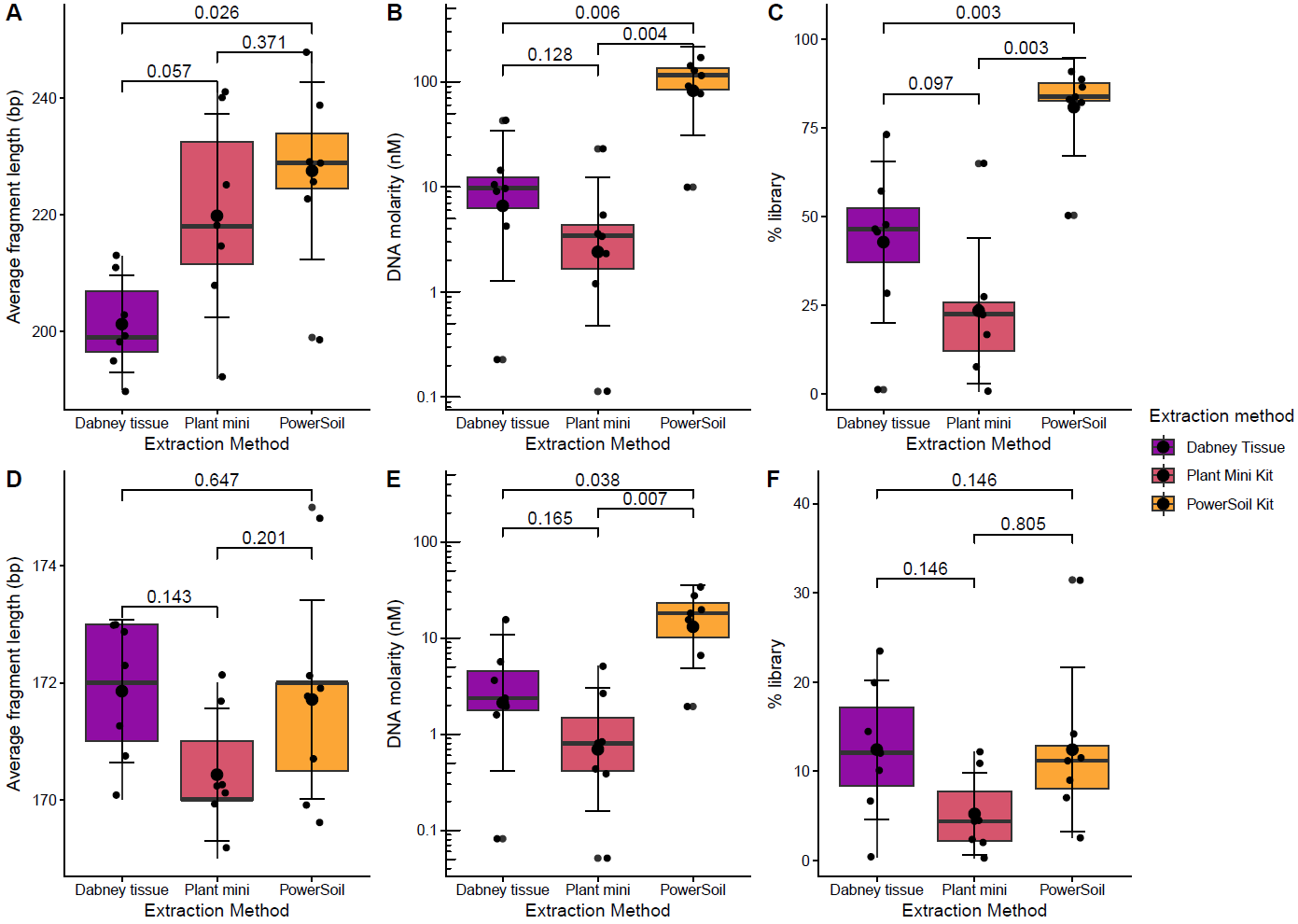


Supplementary Fig. 3. Tapestation peak statistics for the coprolite libraries in the very short fragment range (150-180bp) and main sample range (150-400bp). Tapestation peak statistics for the main sample peak (150-400bp; A-C) and the very short fragment lengths (150-180bp; D-F) of the libraries prior to sequencing. Note that insert length is approximately 150bp shorter than fragment length. (A) Average fragment length of the libraries in the 150-400 bp region. (B) Molarity of the libraries in the 150-400 bp region . (C) % of the libraries represented in the 150-400 bp region. (D) Average fragment length of the libraries in the very short 150-180 bp region. (E) Molarity of the libraries in the very short 150-180 bp region . (F) % of the libraries represented in the very short 150-180 bp region.


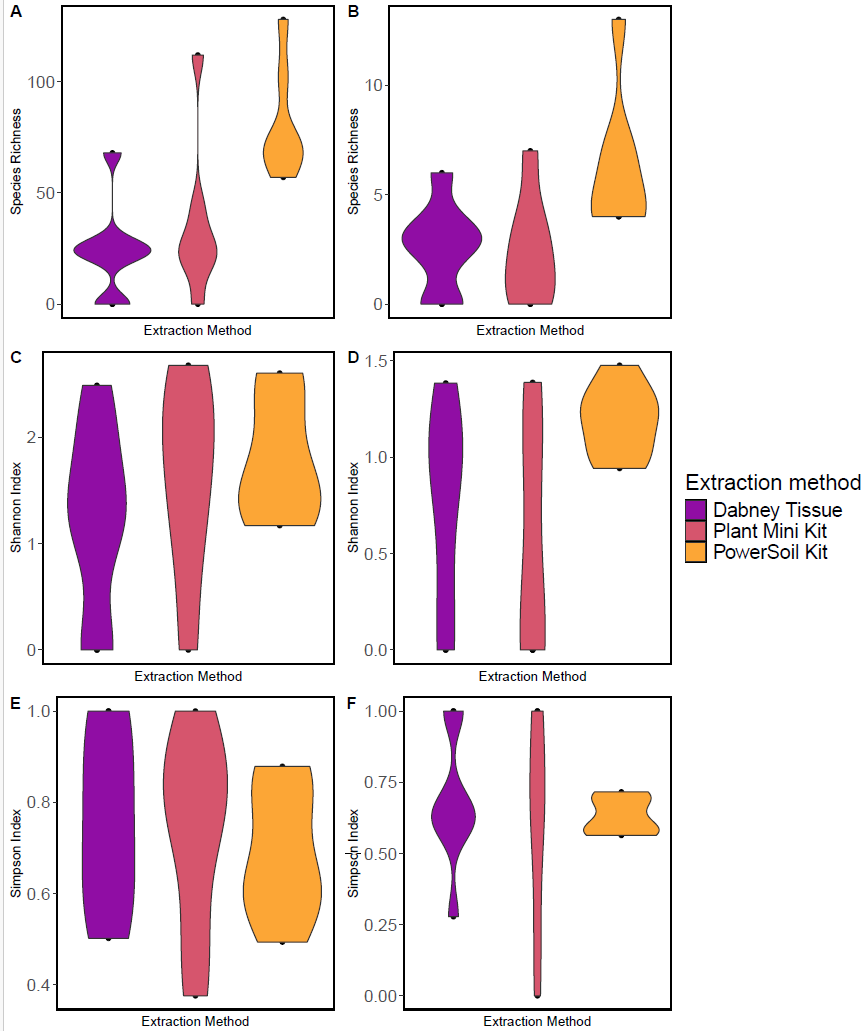


Supplementary Fig. 4. Alpha diversity metrics of the libraries by extraction protocol. A) Species richness in the libraries. B) Plant species richness. C) Shannon index for the whole metagenome. D) Shannon index for plants identified. E) Simpson Index for the whole metagenome. F) Simpson Index for the plant species identified.
